# Multifeature Fusion Neural Network for Oceanic Phenomena Detection in SAR Images

**DOI:** 10.3390/s20010210

**Published:** 2019-12-30

**Authors:** Zhuofan Yan, Jinsong Chong, Yawei Zhao, Kai Sun, Yuhang Wang, Yan Li

**Affiliations:** 1 National Key Lab of Microwave Imaging Technology, Beijing 100190, China; yanzhuofan17@mails.ucas.ac.cn (Z.Y.);; 2 Aerospace Information Research Institute, Chinese Academy of Sciences, Beijing 100190, China; 3 School of Electronics, Electrical and Communication Engineering, University of Chinese Academy of Sciences, Beijing 101408, China

**Keywords:** SAR, deep learning, oceanic phenomena, multifeature fusion, CNN

## Abstract

Oceanic phenomena detection in synthetic aperture radar (SAR) images is important in the fields of fishery, military, and oceanography. The traditional detection methods of oceanic phenomena in SAR images are based on handcrafted features and detection thresholds, which have a problem of poor generalization ability. Methods based on deep learning have good generalization ability. However, most of the deep learning methods currently applied to oceanic phenomena detection only detect one type of phenomenon. To satisfy the requirements of efficient and accurate detection of multiple information of multiple oceanic phenomena in massive SAR images, this paper proposes an oceanic phenomena detection method in SAR images based on convolutional neural network (CNN). The method first uses ResNet-50 to extract multilevel features. Second, it uses the atrous spatial pyramid pooling (ASPP) module to extract multiscale features. Finally, it fuses multilevel features and multiscale features to detect oceanic phenomena. The SAR images acquired from the Sentinel-1 satellite are used to establish a sample dataset of oceanic phenomena. The method proposed can achieve 91% accuracy on the dataset.

## 1. Introduction

Over the ocean, oceanic phenomena affect sea surface roughness [1,2,3,4,5,6]. Synthetic aperture radar (SAR) can estimate the sea surface roughness by backscattering, so various oceanic phenomena can be observed from SAR images, including both natural oceanic phenomena such as oceanic eddies, oceanic fronts, rain cells, and oil spills, and artificial oceanic phenomena, such as ship wakes. The detection of various phenomena using SAR images is one of the key research areas in oceanic applications.

The features of oceanic phenomena in SAR images are affected by environmental conditions and satellite parameters. For example, oceanic eddies appear as bright and dark features in SAR images due to wind direction [7]. Wind speed affects the strength of the features of oceanic phenomena in SAR images [8]. Satellite parameters such as different bands, polarizations, and incidence angles also have an effect on the features of oceanic phenomena in SAR images [9,10,11]. Due to the influence of these factors, the features exhibited by oceanic phenomena are very complicated, which makes the detection of oceanic phenomena difficult.

Traditional methods [12,13,14,15,16,17,18,19,20,21,22] for detecting oceanic phenomena in SAR images are based on handcrafted features and detection thresholds. The features and thresholds used usually need to be designed for a class of oceanic phenomena in a sea area, so the generalization ability is poor. In addition, traditional methods are susceptible to noise interference. It is difficult to efficiently extract features and set appropriate thresholds when there is noise in the image.

In response to the above problems, researchers have applied artificial intelligence methods [23,24,25,26] to the detection of oceanic phenomena in SAR images. In recent years, deep learning methods in artificial intelligence have gradually become mainstream. Image features do not need to be manually modeled, but rather, deep features of the image are extracted through multilevel self-learning, which can effectively address the high degree of feature similarity and large geometric differences in oceanic phenomena. The introduction of deep learning methods to the detection of oceanic phenomena in remote sensing images can greatly improve accuracy. Lima et al. [27] applied convolutional neural networks to realize the effective detection of oceanic fronts in sea surface temperature (SST) images. Lguensat et al. [28] proposed a network called EddyNet, which has an accuracy of 89.83% for oceanic eddies detection in sea surface height (SSH) images. Franz et al. [29] developed a detection framework for oceanic eddies in sea level anomaly (SLA) images. Huang et al. [30,31] proposed an oceanic eddies detection network for SAR images. Its optimal detection accuracy is 95%, which is much higher than the 80% accuracy obtained by traditional methods on the same dataset. Wang et al. [32,33] used a network called inception-v3 for category information detection of oceanic phenomena in SAR images, achieving greater than 90% accuracy, and discussed how to apply these detection results.

Although the deep learning methods have shown excellent performance in the detection of oceanic phenomena in SAR images, there are still some shortcomings. Regarding current research, there is a lack of SAR image datasets of oceanic phenomena that have been annotated by professional experts. In addition, few studies have used deep learning methods to detect oceanic phenomena in SAR images. Previous studies have only detected one type of oceanic phenomenon, or only extracted category information, which does not meet the growing demand.

As a step forward, we use images acquired by the Sentinel-1 satellite to create a sample dataset of oceanic phenomena in SAR images and propose a network called a multifeature fusion neural network (MFNN) to detect oceanic phenomena by fusing multilevel features and multiscale features. Different from the existing methods for oceanic phenomena detection, MFNN realizes the detection of various oceanic phenomena and outlines them. MFNN also improves the detection accuracy of linear oceanic phenomena (such as oceanic fronts and ship wakes) that are difficult to detect in SAR images by improving the extraction of scale features and using weights in the parameter optimization process. We use the sample dataset to train and test the MFNN. The experimental results show that the network can detect the location and class information of multiple oceanic phenomena and achieves an average detection accuracy of 91%, which proves the effectiveness of the network.

The remainder of this paper is organized as follows. In Section 2, we provide a detailed description of the oceanic phenomena detection network—the MFNN. In Section 3, we describe the methods of establishing, expanding, and labeling the SAR image dataset of oceanic phenomena. In Section 4, the experimental results are given and analyzed. The discussions and conclusions are given in Section 5 and Section 6, respectively.

## 2. Multifeature Fusion Neural Network for Oceanic Phenomena Detection in SAR Images

### 2.1. Overview

This paper studies the detection of five types of oceanic phenomena: Oceanic eddies, rain cells, oceanic fronts, ship wakes, and oil spills. Oceanic eddies and rain cells are approximated as evenly distributed surface targets, oceanic fronts and ship wakes are linear targets, and oil spills have both forms. Due to their different physical and geometric properties, various oceanic phenomena exhibit different characteristics in SAR images, and these characteristics have an important impact on oceanic phenomena detection. First, the differences in the backscattering characteristics of oceanic phenomena are small, contributing to poor distinguishability among different oceanic phenomena. Second, the formation of oceanic phenomena is affected by a variety of oceanic elements, which causes similar oceanic phenomena in different sea areas to exhibit different characteristics. Third, there are also huge differences in the shape and scale of similar oceanic phenomena. Finally, there are also situations in which various oceanic phenomena are superimposed.

To account for the weak distinction and changeable characteristics of the oceanic phenomena in SAR images, we extract multilevel features and employ multilevel features for detection. It is difficult to distinguish oceanic phenomena with similar features using only low-level features for detection. However, if only deep-level features are used for detection, oceanic phenomena with smaller scales become easily lost in the background noise during the downsampling process of feature extraction. Therefore, it is necessary to perform multilevel image feature extraction to obtain deep-level features and low-level features.

For the scale difference and superposition of the oceanic phenomena in SAR images, we extract multiscale features and obtain local and global information at different scales. Using only singlescale feature information is not sufficient to detect multiple phenomena with different scales and cannot cope with the problem of superposed oceanic phenomena. Moreover, oceanic phenomena are distributed targets with insignificant features in SAR images, and detection results can become easily disconnected. By extracting multiscale features, it is possible to detect the phenomena from the overall information and avoid detecting phenomena as multiple phenomena due to local information interference.

**Figure 1:**
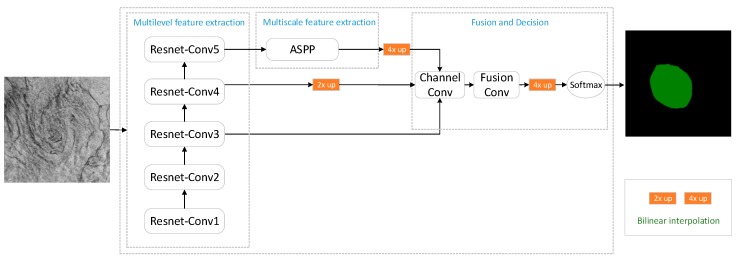
The structure of the multifeature fusion neural network (MFNN).

For the above reasons, we propose the MFNN to detect oceanic phenomena with reference to the DeepLab [34,35,36,37] series and U-net [38], which are effective networks in the field of image detection. The MFNN includes multilevel features extraction, multiscale features extraction, fusion and decision, and parameter optimization modules. The network structure of the MFNN is shown in Figure 1.

The multilevel features extraction module uses ResNet-50 [39] to extract image features. ResNet-conv1~5 in Figure 1 represents the five blocks of ResNet-50. Considering the small number of samples in the dataset, selecting a medium-size network such as ResNet-50 can meet the requirements of multilevel features extraction, effectively limiting the complexity of the model and avoiding over-fitting. Then, atrous spatial pyramid pooling [40] (ASPP) extracts multiscale features. Atrous convolution [41] can reduce information loss when extracting multiscale features while ensuring that the resulting multichannel feature maps have the same resolution. Finally, multilevel features and multiscale features are fused by multiple convolutions, and the fusion results are evaluated by the softmax function to obtain the detection results. The weight parameters of the MFNN need to be trained and tuned before detection applications. Therefore, we use the weighted balance cross-entropy loss function and stochastic gradient descent (SGD) to optimize the MFNN parameters. The following sections detail the various parts of MFNN.

### 2.2. Multilevel Features Extraction

**Figure 2:**
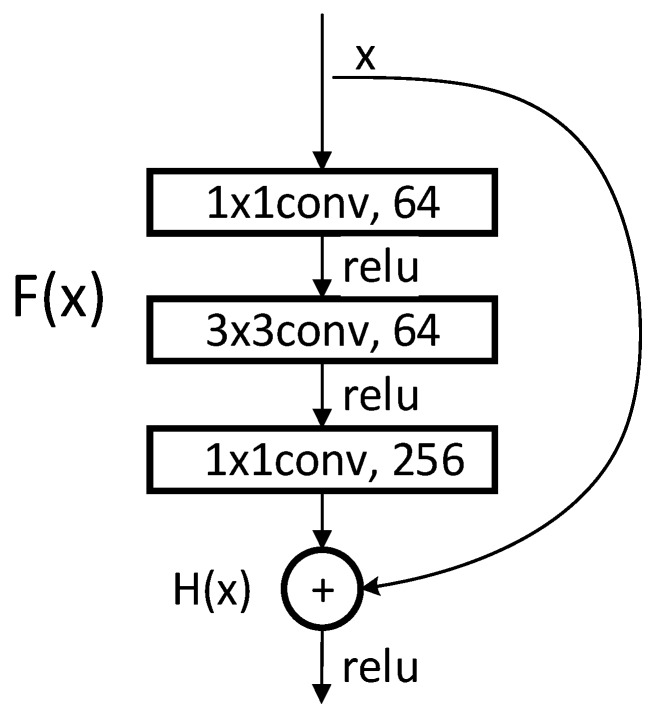
Basic bottleneck module in ResNet-50 [39].

We use ResNet-50 as a multilevel features extraction network. ResNet is a network with deep layers and a low risk of over-fitting. It is connected by multiple residual connection blocks, as shown in Figure 2. By suppressing the problem of gradient disappearance through the residual mechanism, it is possible to construct a deeper network and extract deeper features.

In Figure 2, H(x) represents the combination of feature mapping F(x) and original input x. F(x) represents the main feature learning module, which is usually a concatenation of convolutional layers and activation layers. Express the principle of residual connection as a mathematical form:

(1) H(x)=F(x)+x

Unlike the original ResNet-50, we removed the global average pooling and fully connected layers. The 7×7 convolution kernel with a stride of 2 in Conv1 was replaced with three 3×3 convolution kernels with a stride of 1. This modification reduces the loss of original information in the first layer and makes the feature extraction more stable. The stride of the first 1×1 convolution kernel in Conv3~5 is 2, and the strides of the other 1×1 convolution kernels are 1. Downsampling is performed by convolution between each block to ensure that features at each level are extracted at different information scales. Redundant information is effectively removed, and the required calculations are reduced. The specific parameter setting is shown in Table 1.

In order to achieve a good detection effect, we need to use the high-level feature information and low-level feature information extracted by ResNet-50. To select the best feature set for detection, experiments were performed using different levels of features extracted by the ResNet-50. Experimental results show that the detection using the features of the last three blocks is the best. When using the feature outputs from all five blocks, the detection accuracy does not improve significantly, and some non-target objects are detected as targets. When only the feature outputs of the last two blocks are used, some small-scale oceanic phenomena become undetectable, and some detection results are incomplete. This is because when too many low-level features are used, more noise is introduced. It does not help to improve the detection accuracy, but instead causes some interference targets. When too few low-level features are used, due to lack of detailed information, some small phenomena cannot be detected, and some detection results are incomplete. So after many experiments, this study mainly uses the feature outputs of the two blocks of Conv3 and Conv4 in ResNet-50 and the deep feature output of Conv5. The feature outputs of Conv3 and Conv4 are reserved for the feature fusion module, and the features of the deep feature output are used for further multiscale features extraction.

### 2.3. Multiscale Features Extraction

We use an atrous spatial pyramid pooling module to extract multiscale features. This module is different from the traditional pyramid pooling module in that it uses atrous convolution to extract features. Atrous convolution is a convolution with zero weights in the kernel, which adds an atrous rate parameter to the traditional convolution. The atrous rate can be understood as the sampling step size between the convolution kernel elements, which determines the extent of the convolution kernel receptive field in the image. Because of the atrous rate, the atrous convolution has a greater receptive field than ordinary convolution of the same size. In general, the resolution of the result of the feature map obtained by different atrous convolution is the same.

ASPP uses atrous convolution with different atrous rates arranged in parallel to achieve multiscale features extraction. Since the atrous rate of each channel is different, the receptive field and the scale information of the output feature maps also differ. Because the output feature maps of different channels have the same size, the feature maps can be directly connected together in the channel dimension, eliminating the problem of matching the size during upsampling. When the atrous rate is too large, the information of the output feature maps is almost entirely derived from the zero-padding area, and it will interfere with the result because these areas do not contain any useful information. In order to avoid this problem, we add a global average pooling channel.

**Figure 3:**
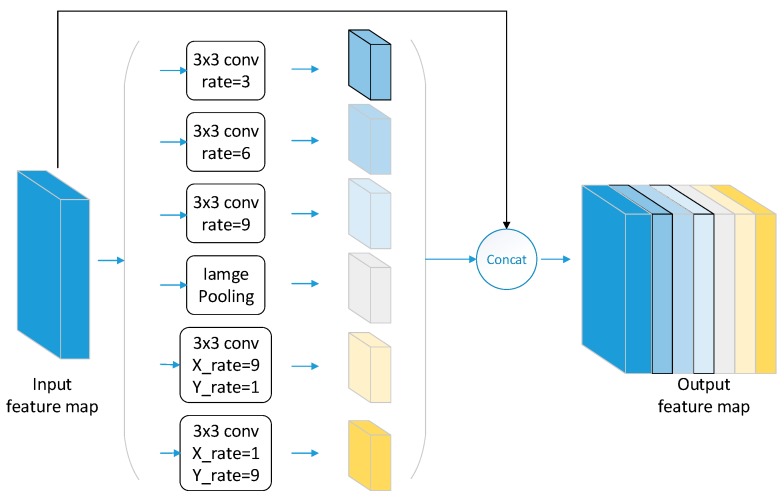
The structure of improved atrous spatial pyramid pooling (ASPP).

A square atrous convolution kernel in ASPP can perform well in extracting features from oceanic phenomena such as rain cells and oceanic eddies, but it will reduce the expression of the features of linear targets, such as ship wakes and oceanic fronts. Therefore, we add two special rectangular atrous convolution modules to ASPP to enhance the feature extraction of linear targets. The improved ASPP module is shown in Figure 3. This module improves the detection accuracy of ship wakes and oceanic fronts, and it reduces the discontinuity probability in linear target detection.

To select the optimal atrous rate of ASPP under our sample dataset, we experimented with square atrous convolution and rectangular atrous convolution under different atrous rate combinations. When the atrous rate is set to {(2, 4, 6), 9×1, 1×9}, the detection accuracy is the highest. Therefore, we set the atrous rate of the three square atrous convolutions to 2, 4, and 6. A rectangular convolution kernel with a horizontal atrous rate X rate of 9 and a vertical atrous rate of 1 and another rectangular convolution kernel with a horizontal atrous rate X rate of 1 and a vertical atrous rate of 9 were set.

### 2.4. Fusion and Decision

**Figure 4:**
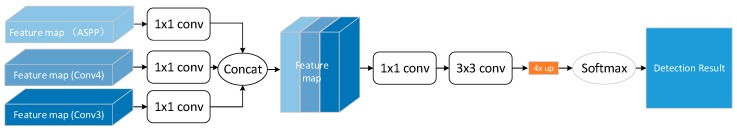
Fusion and decision network structure.

After obtaining multilevel feature maps and multiscale feature maps from ResNet-50 and ASPP, the feature maps are fused to determine the detection results. For this purpose, as shown in Figure 4, the feature maps that are output from Conv3 and Conv4 in ResNet-50 and the feature maps that are output from ASPP are subjected to 1×1 convolution to adjust the channel number. The proportion of different levels of information must be balanced to avoid noise from the underlying information. Then, the channel-adjusted feature maps are concatenated, and one 1×1 convolution and one 3×3 convolution are used to fuse the feature maps. The fusion result image is restored to the original input image size by bilinear interpolation.

We use the softmax function to calculate the result of the detection. Suppose we detect K classes, the original image input is set to x, and the network weight parameters are set to $\theta =(\theta_{1},\theta_{2},\ldots \ldots ,\theta_{n})$. Then, the value at the coordinate (i,j) of the feature map can be represented by a vector $f{\left(x|\theta \right)}_{i,j}$ of length K. The probability that the result at (i,j) belongs to the k-th class is defined as $p_{i,j,k}$. Then, the probability of the corresponding position of the pixel belonging to the k-th class is calculated by using the softmax function:

(2) $$p_{i,j,k}=\frac{e^{f{\left(x|\theta \right)}_{i,j,k}}}{\sum_{k=1}^{K}e^{f{\left(x|\theta \right)}_{i,j,k}}}$$

### 2.5. Parameter Optimization

After determining the MFNN structure, it is necessary to train and optimize the parameters of the MFNN with the training dataset. First, we must define the loss function, which is used to describe the difference between the MFNN outputs and the ground truth of original input images. Class imbalance is a key problem in the actual calculation. Oceanic phenomena that occupy fewer pixels in the images have less influence on the calculation of the loss value, which makes the network insensitive to such phenomena during parameter optimization. This prevents the parameters from being effectively updated in the direction of detecting these phenomena during training, which leads to the detection effect of this kind of phenomenon being unimproved as the number of training iterations increases. Therefore, we use the weighted balance cross-entropy loss function to calculate the loss value during training and to increase the influence of phenomena comprising fewer pixels on the loss value by weighting to solve the problem of class imbalance.

Assume that the detection output image has Q=I×J pixels. Let $Y_{i,j}$ be the ground truth of the pixel at the coordinate (i,j) of the detection output image. If there are K classes for detection, then the cross-entropy loss function can be defined as:

(3) $$C(Y,p)=-\frac{1}{Q}\sum_{i=1,j=1}^{I,J}\sum_{k=1}^{K}Y_{i,j,k}\log(p_{i,j,k})$$

To maintain the balance between different types of oceanic phenomena and to strengthen the influence of classes with a small number of pixels on the loss value, the weighted balance cross-entropy loss function can be obtained by weighting each class:

(4) $$C(Y,p)=-\frac{1}{Q}\sum_{i=1,j=1}^{I,J}\sum_{k=1}^{K}w_{k}Y_{i,j,k}\log(p_{i,j,k})$$

The weight $w_{k}$ can be defined as the ratio of ∑Q and $\sum Q_{k}$. The total number of pixels of the overall sample in the training dataset is defined as ∑Q. The total number of pixels of the k-th class samples in the training dataset is defined as $\sum Q_{k}$. Then, $w_{k}$ can be expressed as:

(5) $$w_{k}=\frac{\sum Q}{\sum Q_{k}}$$

After determining the loss function, the network weights are iteratively optimized by gradient descent according to the loss value. However, the amount of data generated when the entire set of sample images are used to calculate the loss value is too large. Therefore, we use SGD as the parameter optimization algorithm. SGD only randomly selects some of the samples for parameters updating, effectively reducing the complexity of the calculation. If we assume that the number of training samples used in each step is M and the learning rate is η, then the parameter update formula for the SGD is:

(6) $$\theta_{n}{}^{\prime }=\theta_{n}-\eta \frac{\partial \frac{1}{M}\sum_{m=1}^{M}C_{m}(Y,p)}{\partial \theta_{n}}$$

We use the sample dataset of oceanic phenomena in SAR images as the training samples and use the SGD to update the MFNN parameters. When the loss value converges, we obtain the network of optimal parameters for the current sample dataset.

## 3. Establishment of the Sample Dataset of Oceanic Phenomena

### 3.1. Sample Dataset Construction

We select five types of phenomena, oceanic eddies, rain cells, ship wakes, oceanic fronts, and oil spills as objects to be detected. The proposed MFNN performs detection by automatically learning the features of oceanic phenomena using a multilevel network model. Because the process of network learning features needs to be driven by training data, the quality of the training dataset is the key factor that affects the accuracy of the detection results. Therefore, we created a dataset of SAR images containing five oceanic phenomena.

The original dataset of images of oceanic phenomena is derived from SAR images acquired by the Sentinel-1 satellite in 2015–2017. From these images, we identified SAR images containing oceanic phenomena. To better distinguish the image content and to improve the accuracy of phenomena recognition, we enhance the contrast of the images and adjust the gray level of the images to ensure that the oceanic phenomena are prominent. Finally, we cropped the SAR images containing oceanic phenomena. To retain more information in the images, we do not filter the SAR images during the cropping process. The resulting oceanic phenomena dataset is not only rich in physical features such as structure and scale, but also has significant diversity in visual features and texture features.

### 3.2. Sample Dataset Expansion

For detection methods based on deep learning, the number of samples is related to the generalization ability. A sufficient and diverse training dataset is key to the excellent performance of deep learning methods in image detection. However, due to the wide coverage of the ocean, the oceanic phenomena are formed by complex mechanisms, which increases the difficulty of constructing the oceanic phenomena dataset of SAR images. Thus, the number of samples in the dataset is very limited, increasing the risk of network over-fitting.

To satisfy the requirements of network training for data diversity and to achieve satisfactory automatic detection accuracy, we use image expansion methods to expand the training dataset. It increases the image diversity and improves the generalization ability and robustness of the network.

Common image expansion methods include rotation transformation, flip transformation, scaling transformation, and translation transformation. To avoid the correlation between the detection result and the position of the oceanic phenomenon in the image, we use the translation transformation method to simulate the random distribution of phenomena within the images to improve the translation invariance of the MFNN. We rotate the images to change the attitude information, expand the diversity of the images, and make the MFNN more robust against angular features.

Land and islands, which often appear in SAR oceanic images, can introduce problems in the detection of oceanic phenomena. Land and islands can be removed by image pre-processing, but this step takes additional time and significantly reduces the ability to automate system operation. Therefore, we do not use pre-processing but consider land, islands, and ocean background as negative samples during training so that these features are judged as a background class during testing to avoid incorrect detection results.

By randomly cropping, rotating, and stitching the images, we obtained a dataset containing 2000 samples. Each oceanic phenomenon corresponded to 400 images. Furthermore, 200 land and island images and 200 images of oceanic phenomena in other categories were added, bringing the total size of the final dataset to 2400 samples. Finally, considering the retention of image information and the limitations of computing resources, we resized the images of the dataset to 512 × 512 pixels.

### 3.3. Sample Dataset Annotation

**Figure 5:**
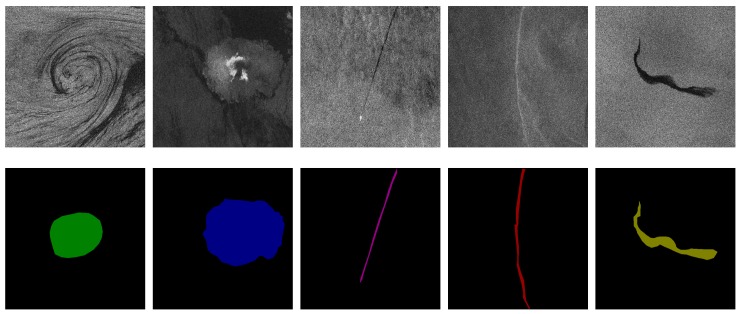
Examples of oceanic phenomena with their corresponding annotation.

After generating the original oceanic phenomena dataset of SAR images, the oceanic phenomena of each SAR image in the dataset must be annotated by professional experts. We take into account the weak boundaries of oceanic phenomena and annotate only the core of oceanic phenomena to avoid introducing noise that affects the accuracy of detection when annotating. As shown in Figure 5, oceanic eddies were annotated in green, rain cells in blue, ship wakes in purple, oceanic fronts in red, and oil spills in yellow. Thereby, an annotated image for each slice image is obtained. The images of oceanic phenomena and the annotated results are collated to construct a complete sample dataset.

## 4. Experiment and Analysis

### 4.1. Experiments on the Single Type of Oceanic Phenomena in SAR Images

**Figure 6:**
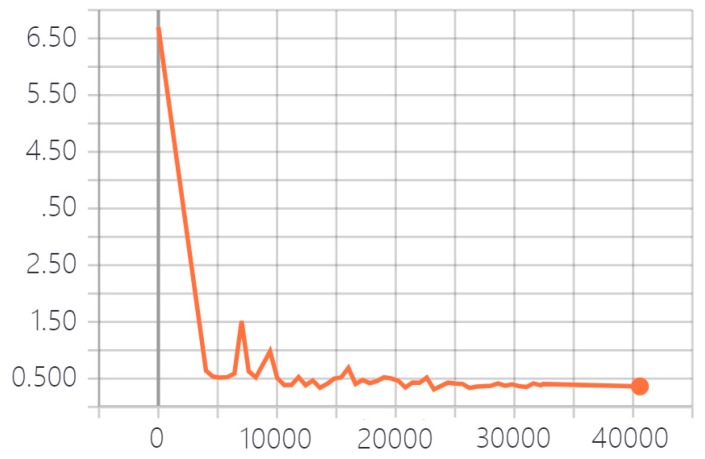
Loss value changes with the steps of iteration.

We provided a detailed introduction to the MFNN in Section 2 and then verified the validity of the network by analyzing actual images. We used the oceanic phenomena dataset to train the MFNN for 40,000 iterations. Figure 6 shows that the loss value of the MFNN no longer changes at approximately 35,000 iterations. Therefore, we used the network obtained through the 32,000th training for testing.

**Figure 7:**
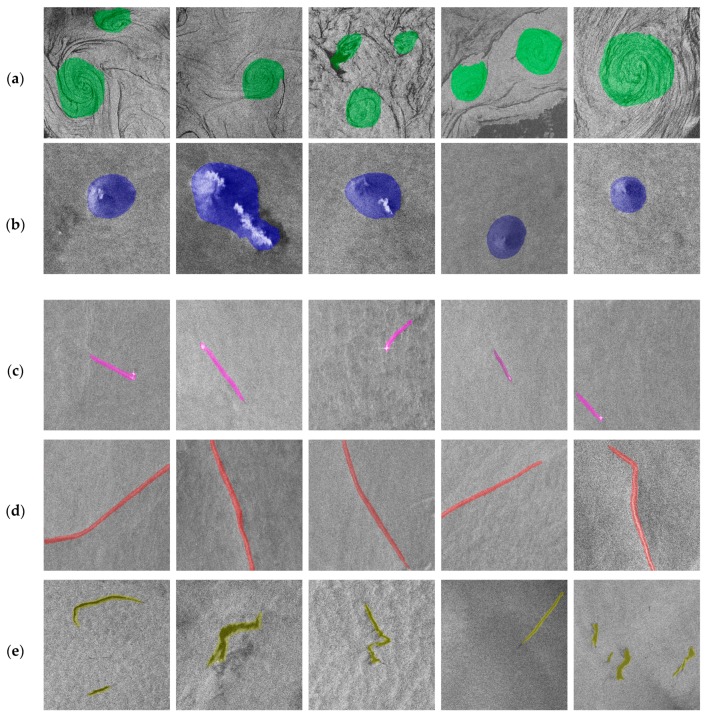
Oceanic phenomena detection results. (**a**) Examples of oceanic eddy detection results. (**b**) Examples of rain cell detection results. (**c**) Examples of ship wake detection results. (**d**) Examples of oceanic front detection results. (**e**) Examples of oil spill detection results.

The MFNN was tested using images from the testing dataset. The correct detection results of the oceanic phenomena in the SAR slice images are shown in Figure 7, in which the green represents oceanic eddies, blue represents rain cells, purple represents ship wakes, red represents oceanic fronts, and yellow represents oil spills. Due to the weak boundaries of oceanic phenomena, the training image dataset only annotated the core region of the oceanic phenomena in the images. Therefore, testing was also based on whether an oceanic phenomenon core region was detected to determine the accuracy of the detection results.

We perform statistical analysis and accuracy calculations on the detection results of various oceanic phenomena. As Table 2 shows, the average accuracy of the test results reached 91%.

**Figure 8:**
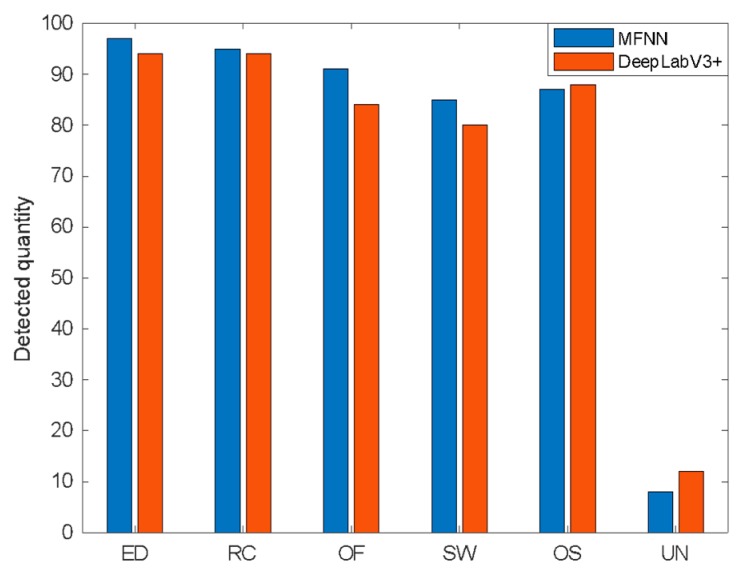
The comparison of two network test results, where **ED** is oceanic eddy, **RC** is rain cell, **OF** is oceanic front, **SW** is ship wake, **OS** is oil spill, and **UN** is the unknown category.

In the research of some existing remote sensing image detection usages, DeepLabV3+ has shown excellent performance and better detection accuracy compared to other networks such as PspNet, SegNet, U-net, FCN, and so on [42,43,44,45,46,47,48]. To demonstrate the superior performance of MFNN, we conducted experiments using MFNN and DeepLabV3+, respectively. The number of correct classifications of each kind of phenomenon detected by DeepLabV3+ and the MFNN is counted, and a histogram is plotted in Figure 8 to compare the results.

**Figure 9:**
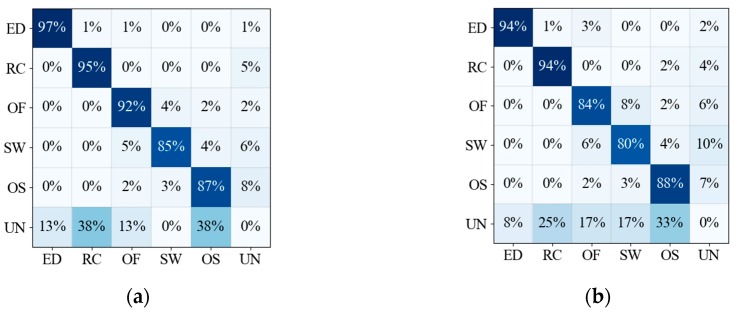
The confusion matrix of the oceanic phenomena detection results, where **ED** is oceanic eddy, **RC** is rain cell, **OF** is oceanic front, **SW** is ship wake, **OS** is oil spill, and **UN** is the unknown category. (**a**) The confusion matrix of the MFNN. (**b**) The confusion matrix of DeepLabV3+.

According to the detection results of DeepLabV3+ and MFNN, the corresponding confusion matrix is shown in Figure 9.

It can be seen that the MFNN has better performance in the detection, and the detection effect on linear targets has been significantly improved. From Table 3, the results show that MFNN is the winner compared with DeepLabV3+ on any metrics (precision, recall, F1, and accuracy).

### 4.2. Experiments on Multiple Types of Oceanic Phenomena in SAR Images

**Figure 10:**
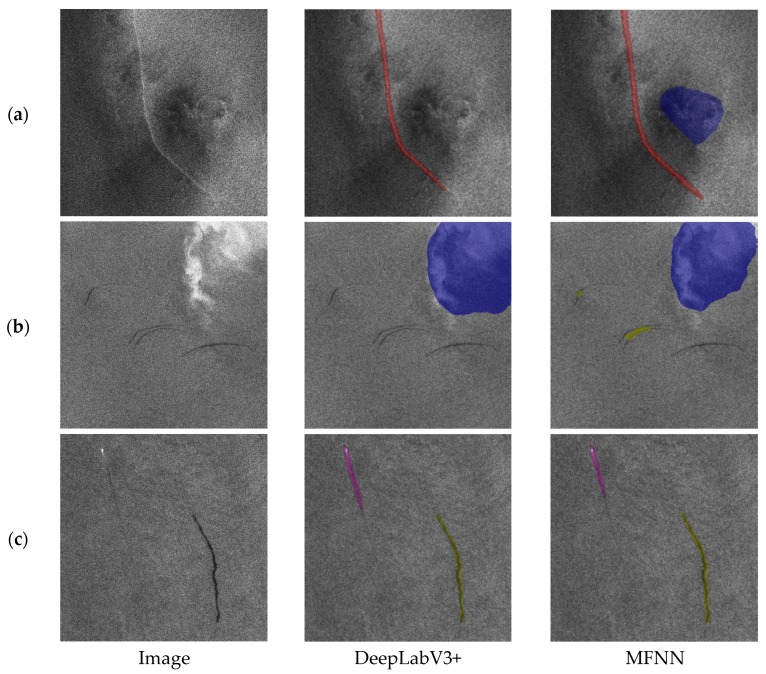
The detection results when multiple types of oceanic phenomena coexist in the image. (**a**) Oceanic front and rain cell. (**b**) Oil spill and rain cell. (**c**) Ship wake and oil spil.

In order to prove the detection ability of MFNN for images of multiple coexisting phenomena, experiments were performed on images with multiple phenomena in the same scene. Due to the limited number of samples, no images of multiple different coexisting oceanic phenomena were added to the training dataset. But as shown in Figure 10, the results show that when multiple phenomena coexist in the image, MFNN can still perform better than DeepLabV3+.

It can be seen that in Figure 10a, compared with DeepLabV3+, MFNN detected a complete rain cell. In Figure 10b, MFNN detected two small-scale oil spills. The performance of the two methods in Figure 10c is similar. The experimental results show that MFNN achieves better performance when multiple phenomena coexist.

**Figure 11:**
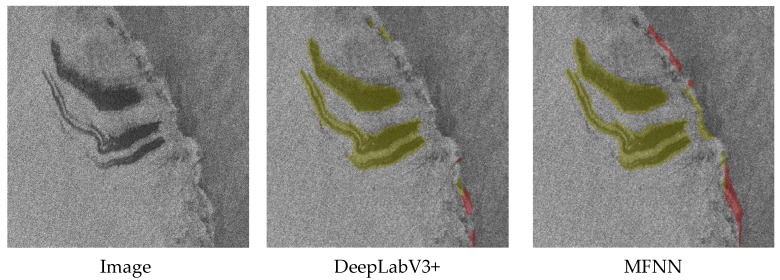
The detection results for atmospheric front that do not exist in the training dataset.

As shown in Figure 11, although we did not include the atmospheric front in the training dataset, our method still detected the atmospheric front as a front. This is because atmospheric fronts and oceanic fronts are both fronts and have similar features. This also illustrates the superior ability of our method for feature extraction. In further research, we will add atmospheric fronts to the training set to further distinguish between atmospheric fronts and oceanic fronts, or improve the accuracy of detecting them as one category.

The above results show that MFNN has good performance in SAR images with multiple types of oceanic phenomena. Due to the limitation of samples, the precision, recall, F1 and accuracy of this section are not considered. In future research, we will expand the dataset of SAR images with multiple types of oceanic phenomena to further verify and improve detection performance.

## 5. Discussion

### 5.1. Influence of Network Structure on the Detection Results

After many experiments, we only use the features from partial levels obtained in ResNet-50 when designing the network structure, and multiscale features extraction is only carried out for the highest-level features. Using different combinations of features from different levels will have different detection effects. Multiscale feature extraction for multiple-level features also has an impact on the detection effect. So we will further explore new structures and try to introduce new structures to utilize other low-level information, which may have different effects.

When MFNN detects multiple types of oceanic phenomena coexisting or superimposing in one image, some phenomena cannot be detected. Therefore, improving the network structure is necessary in order to improve detection accuracy and extract information capable of distinguishing more phenomena.

### 5.2. Influence of the Dataset on the Detection Results

We trained the MFNN using an image-expanded sample dataset. When expanding the image dataset, the number of samples required for training the network and the methods for image expansion should be comprehensively considered to avoid incorrect detection results. When we annotated the slice images, only the core regions of oceanic phenomena in the images were annotated, and this region was used to represent the entire oceanic phenomenon. This procedure may introduce subjective factors that affect annotations. In future research, we plan to develop an improved method to annotate images to increase the accuracy of the results.

We currently use images from Sentinel-1 for MFNN training and testing. All parameters are optimized according to the Sentinel-1 image dataset, and thus, the current detection network only performs well when Sentinel-1 images are used as input. Because the features of oceanic phenomena in SAR images are highly related to satellite parameters such as polarization and band parameters, the features learned by the network through the Sentinel-1 satellite training dataset cannot be directly applied to the images from other satellites. If the network is to be used to analyze images acquired from other satellites, the hyperparameters should be reselected, and the network should be trained and tuned using the corresponding satellite image dataset.

Only five types of oceanic phenomena, namely oceanic eddies, rain cells, oceanic fronts, ship wakes, and oil spills, were included in the current study for analysis by the MFNN. In future research, we will construct a complete dataset and improve the network structure in order to detect sea ice, internal waves, and other oceanic phenomena.

## 6. Conclusions

To solve the problems of low efficiency in the detection of oceanic phenomena in SAR images, we propose a detection network called the MFNN based on ResNet-50 and ASPP. Different from the existing methods in the current research, MFNN can detect the location and category information of multiple oceanic phenomena by extracting and fusing the multilevel features and multiscale features of SAR images. Moreover, by improving multiscale feature extraction on the network structure and adding weights during parameter optimization, it makes the detection accuracy of linear oceanic phenomena, such as oceanic fronts and ship wakes, better than other detection methods.

We constructed a sample dataset using SAR images from the Sentinel-1 satellite and used this dataset to train and test the MFNN. The experimental results show that the MFNN proposed in this paper realizes the detection of five types of oceanic phenomena, namely, oceanic eddies, rain cells, oceanic fronts, ship wakes, and oil spills in SAR images and obtains an average detection accuracy of 91%.

## Figures and Tables

**Table 1:** ResNet-50 network architecture parameter settings.

Layer Name	Network	Output Size (Channel × Height × Wide)
Conv1	{3 × 3 conv, stride 1, 64} × 3 3 × 3 max pool, stride2	64 × h/2 × w/2
Conv2	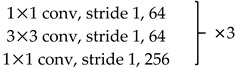	256 × h/2 × w/2
Conv3	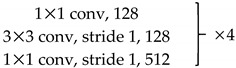	512 × h/4 × w/4
Conv4	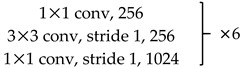	1024 × h/8 × w/8
Conv5	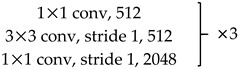	2048 × h/16 × w/16

**Table 2:** Statistics of oceanic phenomena detection results.

Phenomenon	Training Quantity	Testing Quantity	Correction Quantity	Accuracy
Oceanic eddy	300	100	97	97%
Rain cell	300	100	95	95%
Oceanic front	300	100	91	91%
Ship wake	300	100	85	85%
Oil spill	300	100	87	87%
Total	1500	500	455	91%

**Table 3:** Performances of different methods.

Method	Precision (%)	Recall (%)	F1 (%)	Accuracy (%)
DeepLabV3+	91.1	88	89.5	88
MFNN	93.8	91	92.4	91
